# *Chlamydia pneumoniae* Infection in Atherosclerotic Lesion Development through Oxidative Stress: A Brief Overview

**DOI:** 10.3390/ijms140715105

**Published:** 2013-07-19

**Authors:** Marisa Di Pietro, Simone Filardo, Fiorenzo De Santis, Rosa Sessa

**Affiliations:** Department of Public Health and Infectious Diseases, “Sapienza” University, Rome 00185, Italy; E-Mails: marisa.dipietro@uniroma1.it (M.D.P.); filasimo@libero.it (S.F.); fiorenzo.desantis@uniroma1.it (F.D.S.)

**Keywords:** *C. pneumoniae*, atherosclerosis, oxidative stress, inflammation

## Abstract

*Chlamydia pneumoniae*, an obligate intracellular pathogen, is known as a leading cause of respiratory tract infections and, in the last two decades, has been widely associated with atherosclerosis by seroepidemiological studies, and direct detection of the microorganism within atheroma. *C. pneumoniae* is presumed to play a role in atherosclerosis for its ability to disseminate via peripheral blood mononuclear cells, to replicate and persist within vascular cells, and for its pro-inflammatory and angiogenic effects. Once inside the vascular tissue, *C. pneumoniae* infection has been shown to induce the production of reactive oxygen species in all the cells involved in atherosclerotic process such as macrophages, platelets, endothelial cells, and vascular smooth muscle cells, leading to oxidative stress. The aim of this review is to summarize the data linking *C. pneumoniae*-induced oxidative stress to atherosclerotic lesion development.

## 1. Introduction

Atherosclerosis, a major public health problem in developed countries, is a chronic inflammatory disease of multifactor etiology, characterized by endothelial injury, accumulation of monocytic cells, and increased secretion of mediators of inflammation, such as interleukin (IL)-1, IL-6, and tumor necrosis factor (TNF)-α [1].

In addition to the traditional cardiovascular risk factors, in the last two decades, several infectious agents, such as cytomegalovirus, *Helicobacter pylori*, periodontal pathogens, and *Chlamydia pneumoniae*, have been implicated in the pathogenesis of atherosclerosis [2]. *C. pneumoniae* has been considered as the most plausible additional risk factor for atherosclerosis since it is the sole viable pathogen detected in the atherosclerotic plaque [3–6]. Moreover, *C. pneumoniae* is able to multiply and persist within vascular cells and to induce the chronic inflammatory state underlying atherosclerosis [7].

Mainly, two *C. pneumoniae* virulence factors may be involved in atherogenesis: chlamydial lipopolysaccharide (LPS) and chlamydial heat shock protein-60 (cHSP60). LPS, a major chlamydial antigen able to activate an acute inflammatory response, may accelerate foam cell formation and induce platelet activation. Chlamydial HSP60, classically produced during chronic chlamydial infection and capable of activating innate immune and inflammatory responses, is responsible for endothelial dysfunction and proliferation of vascular smooth muscle cells (VSMCs) [8–10].

Over the last few years, a growing body of evidence has shown that oxidative stress, resulting from the imbalance between the production of reactive oxygen species (ROS), such as superoxide anion, hydroxyl radical, and nitric oxide, and the activity of antioxidant systems, is implicated in atherogenesis. However, the cellular events of oxidative stress on vascular wall are very complex and involve several regulatory proteins and enzymes [11,12].

*C. pneumoniae* infection has been shown to induce ROS production in all the cells involved in the atherosclerotic process such as macrophages, platelets, endothelial cells, and VSMCs leading to oxidative stress [13–16].

The aim of this review is to summarize the data linking *C. pneumoniae*-induced oxidative stress to the atherosclerotic lesion development.

## 2. *C. pneumoniae* Infection and Atherosclerosis

*C. pneumoniae* is a widespread respiratory pathogen that causes sinusitis, pharyngitis, and pneumonia. The majority of infections are often asymptomatic and the exposure to *C. pneumoniae* is extremely common; epidemiological studies indicate that anti-*C. pneumoniae* antibody prevalence is 50% by the age of 20 and increases with increasing age [17].

*C. pneumoniae* is presumed to play a role in the pathogenesis of atherosclerosis for its ability to systematically disseminate from the lungs through peripheral blood mononuclear cells (PBMC) and to localize in several tissues, including arteries [18–21].

**Figure 1:**
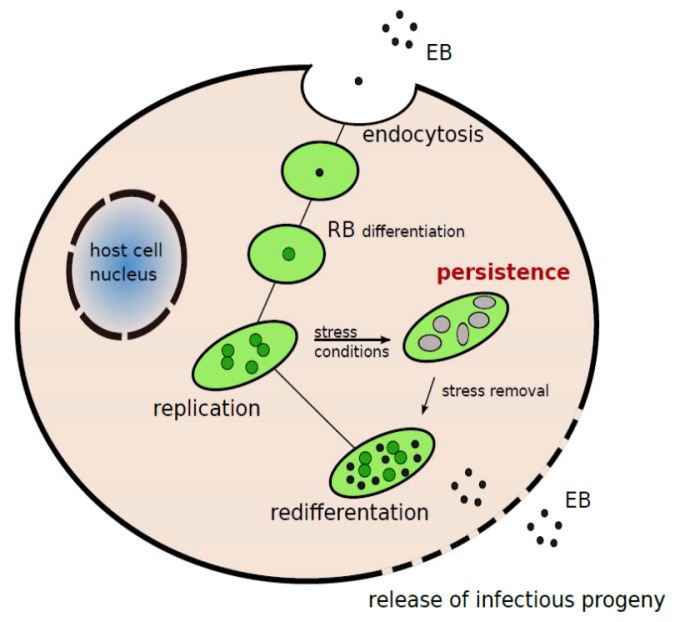
Schematic representation of *C. pneumoniae* developmental cycle. Infectious but metabolically inactive EB enters the host-cell membrane via endocytosis; EB transforms in the replicative and metabolically active RB; RB redifferentiates into EB, which is released by the host-cell via lysis. In the presence of IFN-γ, penicillin G, or other stressful conditions, intracellular *C. pneumoniae* generates a non-infectious persistent form.

*C. pneumoniae,* an intracellular obligate bacterium, has a unique developmental cycle involving two distinct functional and morphological forms: the elementary body (EB) and the reticulate body (RB) (Figure 1). The EB is the metabolically inert and infectious form of the microorganism, capable of transient extracellular survival, whereas the RB is the intracellular replicative but not infectious form. The developmental cycle is initiated by attachment and entry of the infectious EB into the host-cell followed by the transformation of EB to RB, RB division by binary fission, and finally differentiation of RB back to EB, which is released from the host-cell by lysis [22]. *C. pneumoniae* fails to complete its developmental cycle when starved for nutrients, such as iron, or when exposed to certain antibiotics, such as penicillin, or cytokines, such as Interferon (IFN)-γ [23–25]. Under these conditions, *C. pneumoniae* generates enlarged and morphologically aberrant RBs called persistent forms which can remain viable but non-infectious inside the host-cell for a long time; they are inherently more suited to evade the host immune response and completely refractory to antibiotic treatment, which makes chlamydial eradication difficult [7,26]. This may explain the complete failure of large randomized clinical trials (the Azithromycin in Coronary Artery Disease: Elimination of Myocardial Infection with Chlamydia, ACADEMIC, the Weekly Intervention with Zithromax Against Atherosclerotic-Related Disorders, WIZARD, the Azithromycin and Coronary Events Study, ACES, the CLARIthromycin for patients with stable CORonary heart disease, CLARICOR, and the PRavastatin Or atorVastatin Evaluation and Infection Therapy-Thrombolysis In Myocardial Infarction, PROVE IT-TIMI) in showing any benefit of anti-chlamydial treatment [27]. In addition, other factors, such as the lack of markers of persistent chlamydial infection and the enrolment of patients with advanced coronary artery disease, should also be considered [2]. As a result, persistent chlamydial forms may act as a chronic stimulus in the perpetuation of vascular inflammation, thus exacerbating the atherosclerotic process [2,27].

The first suggestion that *C. pneumoniae* may be associated with atherosclerotic cardiovascular diseases was proposed in 1988 by Saikku *et al.* [28]. They showed that patients with acute myocardial infarction and chronic heart disease had more frequently (68%) anti-*C. pneumoniae* antibodies than controls (17%). Since then, several studies (cross-sectional, case-control, or retrospective) have confirmed the association between serological evidence of *C. pneumoniae* infection and atherosclerotic cardiovascular disease (CVD) although others (prospective studies) have failed to demonstrate such association [29–34]. The main limitation of these studies is the difficulty to identify differences in seropositivity between patients and controls, since a large part of the population has pre-existing IgG antibodies from previous exposure [35].

Further evidence that *C. pneumoniae* might play a role in atherosclerosis came from studies in which the microorganism has been detected in atherosclerotic lesions of coronary and carotid arteries and aneurysm of abdominal aorta but not in healthy arteries. By polymerase chain reaction, *C. pneumoniae* DNA has been detected in atherosclerotic plaques; by immunohistochemistry, *C. pneumoniae* has been found in macrophages, endothelial cells, and smooth muscle cells within atherosclerotic lesions; by electron microscopy, *C. pneumoniae* has been shown in atheroma-associated foam cells [36–42]. However, wide variability in *C. pneumoniae* detection exists as a result of a lack of standardized methods [35].

Even more important are *in vivo* and *in vitro* studies showing the atherogenic role of *C. pneumoniae. In vivo* studies have demonstrated that *C. pneumoniae* infection may accelerate the progression of atherosclerotic lesion in hyperlipidemic animal models suggesting that this microorganism is a co-risk factor with hyperlipidaemia and that the atherogenic effects of *C. pneumoniae* are contingent on the vascular response to hyperlipidaemia [43–47]. In particular, recent studies have shown that, in hypercholesterolemic rabbit models, GroEL1 (also known as cHSP60) administration enhanced fatty streak formation and macrophage infiltration in atherosclerotic plaques, which may be mediated by elevated lectin-like oxidized low-density lipoprotein receptor (LOX)-1 expression [48,49].

Importantly, mouse models have provided the evidence that *C. pneumoniae* is able to disseminate via bloodstream to the vasculature and to multiple organs. In particular, it has been showed that intranasal inoculation of *C. pneumoniae* was followed initially by recovery of the microorganism within lungs, PBMCs, heart, and later into the brain [50–53], demonstrating also the presence of persistent chlamydial forms.

Chlamydial DNA in PBMCs has also been demonstrated in patients with CVD by several studies [54–56]. Circulating infected mononuclear cells have been considered as a means by which *C. pneumoniae* can induce a chronic systemic inflammation contributing to the development and progression of CVD.

*In vitro* studies have shown the ability of *C. pneumoniae* to infect and multiply within atheroma-associated cell-types, resulting in various pro-atherosclerotic effects [57–59]. Infection of monocytes with *C. pneumoniae* increases adherence of infected monocytes to endothelial cells and accelerates foam cell formation [60–64]. Furthermore, the multiplication of *C. pneumoniae* inside monocytes or macrophages triggers the production of pro-inflammatory cytokines such as IL-1α, IL-6, monocytes chemoattract protein-1 (MCP-1), macrophage inflammatory protein 1α, and IL-12, promoting lesion progression [65,66]. Moreover, *C. pneumoniae* has been observed to activate macrophages stimulating TNF-α and matrix metalloproteinase (MMP) expression, which may contribute to plaque weakening and subsequent rupture [67]. Recently, our study has shown the possible involvement of IL-17A in *C. pneumoniae* induced foam cell formation [68].

Infection of endothelial cells by *C. pneumoniae* results in enhanced adherence and migration of leukocytes into the vascular wall contributing to the inflammatory state. This occurs through increased nuclear factor kappa-B (NF-κB)-mediated secretion of IL-1, IL-8, and MCP-1 paralleled by expression of adhesion molecules (endothelial-leukocyte adhesion molecule-1, ELAM-1, intercellular adhesion molecule-1, ICAM-1, and vascular cell adhesion molecule-1, VCAM-1) [49,69–72]. *C. pneumoniae* infection of endothelial cells can also trigger VSMCs proliferation through the induction of human heat shock protein 60 (hHSP60) and stimulation of the mitogenic activity of platelet-derived growth factor (PDGF). There is also evidence that *C. pneumoniae* infection in endothelial cells promotes the secretion of plasminogen activator inhibitor-1 (PAI-1) inducing platelet activation, which would further contribute to advanced plaque progression [73–76].

Lastly, infection of VSMCs by *C. pneumoniae* induces the production of IL-6, basic fibroblast growth factor (bFGF), and MMP, via NF-κB activation, contributing to plaque destabilization, and MCP-1 release through toll-like receptor 2 (TLR-2), promoting monocyte migration into intima [77,78].

## 3. *C. pneumoniae* Induces Oxidative Stress in Vascular Cells

**Figure 2:**
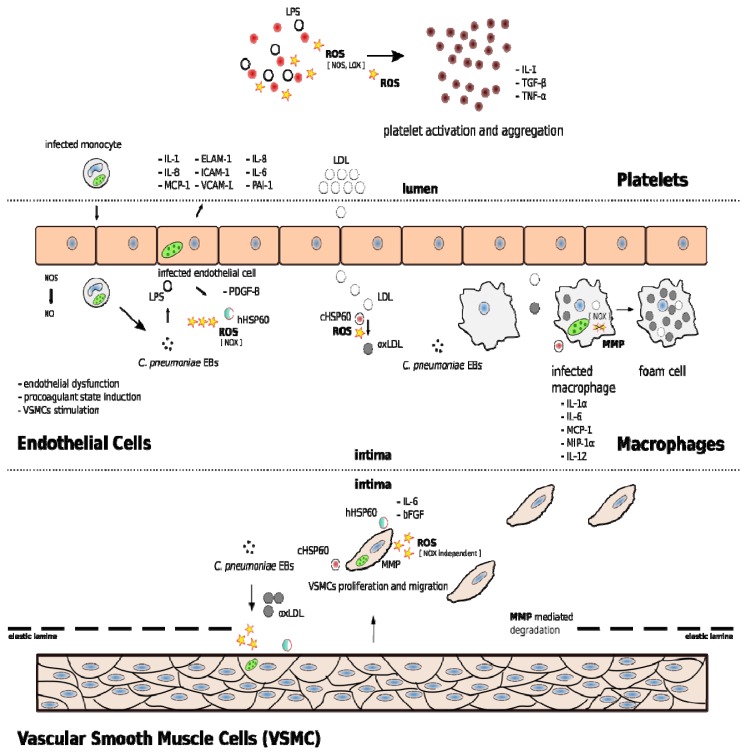
Schematic representation of the cellular events linking *C. pneumoniae*-induced oxidative stress to atherosclerotic lesion development. In platelets, *C. pneumoniae* contributes to platelet activation and aggregation; in endothelial cells, *C. pneumoniae* leads to endothelial dysfunction and induces an increased surface expression of hHSP60; in macrophages, *C. pneumoniae* induces the oxidation of LDL and the uptake of oxLDL, leading to foam cell formation and cHSP60 stimulates macrophages to synthetize MMP; in VSMCs, *C. pneumoniae* enhances cell proliferation and migration.

Emerging evidence have suggested that *C. pneumoniae* infection increases ROS production in all the cells involved in atherosclerosis, such as macrophages, endothelial cells, platelets, and VSMCs, leading to oxidative stress (Figure 2).

Classically, ROS in macrophages play an important role in host defense by killing the invading microorganisms [79]. Nevertheless, by modulating cellular redox balance *C. pneumoniae* is able to survive within macrophages [13,80], generating persistent chlamydial forms. Consequently, macrophages may act as a reservoir of *C. pneumoniae* sustaining chronic infection [81–84].

Moreover, *C. pneumoniae* infection in macrophages, considered as a hallmark of atherosclerosis, may induce oxidative stress. Specifically, *C. pneumoniae* has been shown to induce monocytes to oxidize LDL through the NADPH oxidase-mediated release of superoxide anion (O^2−^) [13]. Furthermore, *C. pneumoniae* infection may also promote the accumulation of LDL into macrophages, partly by increasing the expression of lipoprotein lipase via LPS and by dysregulating receptors involved in cholesterol efflux via nuclear receptor Peroxisome Proliferator-Activated Receptor (PPAR)-γ [85,86]. In accordance with these findings, we have recently observed that resveratrol, a powerful antioxidant, may prevent *C. pneumoniae* induced foam cell formation by decreasing superoxide anion-mediated LDL oxidation and regulating cholesterol efflux into macrophages [68].

Some lines of evidence have also suggested that *C. pneumoniae*-induced oxidative stress in macrophages may contribute indirectly to the progression and destabilization of atherosclerotic plaque. Specifically, *C. pneumoniae* infection augments cell death induced by the accumulation of oxLDL in macrophages, accelerating the formation of atherosclerotic lipid-rich core [87] and worsening vascular inflammation.

Interestingly, *C. pneumoniae*-induced ROS overproduction has also been demonstrated in platelets: *C. pneumoniae* LPS induces the production of ROS through Nitric Oxide synthase (NOS) and lipoxygenase (LOX) pathways, and the activation of protein kinase C [14,88], contributing to LDL oxidation, platelet activation and, consequently, thrombotic vascular occlusion during acute coronary events [89].

*C. pneumoniae*-induced oxidative stress in platelets may also contribute to adhesion of monocytes/macrophages and proliferation and migration of VSMCs. In fact, further studies on *C. pneumoniae* interaction with platelets showed the ability, of this microorganism, to stimulate the secretion of mediators of inflammation such as IL-1, tumor growth factor (TGF)-β, and TNF-α [90,91].

Noticeably, *C. pneumoniae* infection induces also oxidative stress in endothelial cells and VSMCs, promoting endothelial dysfunction and cell migration and proliferation respectively.

It is well known that endothelial dysfunction, characterized by altered endothelium-mediated vasodilation, increased vascular reactivity and platelet activation, is an early event in atherosclerosis and it is due in large part to oxidative stress and reduced endothelial cell nitric oxide bioavailability [92].

*C. pneumoniae*-mediated oxidative stress may induce endothelial dysfunction through three mechanisms. Firstly, cHSP60 significantly increases superoxide anion production and decreases nitric oxide levels [93]. A further finding supporting the *C. pneumoniae*-mediated dysregulation of ROS-related enzymes comes from a recent study that has demonstrated the increased ROS production in infected endothelial cells through the up-regulation of NADPH oxidase (NOX-2 and NOX-4) and down-regulation of superoxide dismutase-1 (SOD-1) and thioredoxin-1 (TRX-1) [16].

Secondly, *C. pneumoniae*-mediated oxidative stress in endothelial cells induces an increased surface expression of hHSP60. In fact, antioxidant treatment significantly reduced hHSP60 expression in response to *C. pneumoniae* infection [16]. In this regard, Wick *et al.* [94] first suggested that the autoimmune reactions against hHSP60 may play a critical role in atherogenesis since both chlamydial and human HSP60 might mimic the ability of *C. pneumoniae* to stimulate the activation of vascular cells, leading to vascular endothelial injury.

Thirdly, *C. pneumoniae*-mediated oxidative stress contributes to endothelial dysfunction through vascular inflammation. Specifically, *C. pneumoniae* has been shown to promote endothelial cell necrosis and, as a result, to enhance the inflammatory effect of oxLDL [95]. A recent study has also shown that oxLDL-induced inflammation may be related to elevated levels of LOX-1, mediated by the phosphoinositide 3-kinase-Akt signaling pathway, endothelial NO synthase activation, NOX-mediated ROS production and mitogen activated protein kinase (MAPK) activation in GroEL1-stimulated human coronary artery endothelial cells [48].

Finally, in VSMCs, *C. pneumoniae* induces the production of ROS that restricts its replication. Under these conditions, *C. pneumoniae* may generate persistent forms that, in turn, aggravate chronic vascular inflammation. Limited growth of *C. pneumoniae* in VSMCs may be due to the fact that ROS production appears to occur independently of NADPH-oxidase activity and myeloperoxidase [96].

In addition, several studies have demonstrated that *C. pneumoniae* infection in VSMCs promotes the uptake of oxLDL, increasing cell proliferation, migration, and adhesion from media to intima through the induction of hHSP60 expression and the activation of MAPK and toll-like receptor 4 (TLR4) pathways [97,98]. Specifically, *C. pneumoniae* HSP60 activated p44/42 MAPK and increased TLR4 mRNA expression [15,99].

## 4. Antioxidant Strategies in *C. pneumoniae*-Mediated Atherosclerosis

*C. pneumoniae*-induced oxidative stress and inflammation are thought to contribute to the initiation, progression and rupture of lipid-rich vascular lesion, and, hence, several treatment strategies have been examined to reduce or prevent them.

Curcumin and resveratrol have been shown to reduce ROS production in *C. pneumoniae* infected THP-1 cells by inhibiting protein kinase C, a trigger of NOX activity, and the assembly of NOX subunits [100]. A further example includes the reduced ROS production in Chlamydia-primed human monocytes by cyclooxygenase (COX)-2 inhibitors [101].

Some studies have suggested that statins, such as simvastatin, cerivastatin, and fluvastatin, may reduce vascular inflammation induced by *C. pneumoniae* in macrophages, endothelial cells and VSMCs [71,102,103].

Interestingly, statins are also well known to reduce oxidative stress [104], since they inhibit oxidants formation by reducing NOX-dependent ROS production, increasing NO availability and stimulating antioxidant defense mechanisms [104]. In fact, PROVE IT-TIMI and a number of subsequent studies [105,106] highlighted the benefit of statin therapy in reducing cardiovascular events [107]. Therefore, more research is helpful to evaluate if statins, as well as other compounds such as ACE inhibitors, may act as antioxidants in *C. pneumoniae*-mediated CVD, even though a better understanding of the interaction between *C. pneumoniae* and the host would be needed to identify specific proteins of persistent chlamydial forms for therapeutic purposes.

## 5. Conclusions

Based on the evidence above described, *C. pneumoniae* interaction with vascular cells results in an imbalance in cell redox state and, consequently, induces oxidative stress responsible partly for the typical pathological changes of atherosclerotic plaques. In particular, *C. pneumoniae*-induced oxidative stress may be involved in both the early stages of atherosclerosis, by promoting macrophage-derived foam cell formation and endothelial dysfunction, and the late stages, by stimulating platelet activation and VSMCs migration and proliferation. In addition, *C pneumoniae*, generating persistent forms and stimulating cytokine production, is able to exacerbate vascular inflammation. Hence, *C. pneumoniae* infection in vascular cells may have a critical role in the vicious cycle between oxidative stress and inflammation in relation to atherosclerosis. As a result, oxidative stress and inflammation induced by *C. pneumoniae* synergize to accelerate atheroma formation and progression, thus increasing risk for vascular disease.
